# Molecular Identification of Cervical Microbes in HIV-Negative and HIV-Positive Women in an African Setting Using a Customized Bacterial Vaginosis Microbial DNA Quantitative PCR (qPCR) Array

**DOI:** 10.1128/spectrum.02229-21

**Published:** 2022-06-01

**Authors:** Ongeziwe Taku, Harris Onywera, Zizipho Z. A. Mbulawa, Charles B. Businge, Tracy L. Meiring, Anna-Lise Williamson

**Affiliations:** a Division of Medical Virology, Department of Pathology, Faculty of Health Sciences, University of Cape Town, Cape Town, South Africa; b Institute of Infectious Disease and Molecular Medicine, University of Cape Town, Cape Town, South Africa; c Centre for Healthcare-Associated Infections, Antimicrobial Resistance and Mycoses (CHARM), National Institute for Communicable Diseases, Division of the National Health Laboratory Service, Johannesburg, South Africa; d Research, Innovations, and Academics Unit, Tunacare Services Health Providers Limited, Nairobi, Kenya; e Research and Innovation, Mount Kenya University, Thika, Kenya; f UCT-MRC Gynaecological Cancer Research Centre, University of Cape Town, Cape Town, South Africa; g National Health Laboratory Service, Nelson Mandela Academic Hospital, Mthatha, South Africa; h Department of Laboratory Medicine and Pathology, Walter Sisulu University, Mthatha, South Africa; i Department of Obstetrics and Gynaecology, Nelson Mandela Academic Hospital, Mthatha, South Africa; j Department of Obstetrics and Gynaecology, Faculty of Health Sciences, Walter Sisulu University, Mthatha, South Africa; University of Utah and ARUP Laboratories

**Keywords:** HIV, cervical microbes, bacterial vaginosis (BV), sexually transmitted infection (STI), emerging sexually transmitted pathogen (pathobiont), African women, bacterial vaginosis microbial DNA qPCR array

## Abstract

Bacterial vaginosis (BV) is a common polymicrobial vaginal disorder that is associated with sexually transmitted infections (STIs), including HIV. Several studies have utilized broad-range 16S rRNA gene PCR assays with sequence analysis to characterize cervicovaginal bacterial communities of women with healthy and diseased conditions. With the high burden of BV and STIs among African women, there is a need for targeted PCR assays that can rapidly determine the true epidemiological profile of key cervical microbes, including BV-associated bacteria, and a need to explore the utility of such assays for microbiological diagnosis of BV. Here, we used a taxon-directed 16S rRNA gene quantitative PCR (qPCR) assay to examine the prevalences and determinants of specific cervical microbes among African women with and without HIV infection. Cervical samples were collected using a cytobrush from 162 women (aged ≥30 years) attending a community-based clinic in Eastern Cape, South Africa. The samples were screened for specific microbes (i.e., STIs, emerging sexually transmitted pathogens [pathobionts], and BV-associated bacteria) using a customized bacterial vaginosis microbial DNA qPCR array. Statistical analyses were performed using GraphPad Prism v6.01. Chi-square/Fisher’s exact tests were used to evaluate the determinants associated with specific cervical microbes. Only 145 women had any detectable microbes and were included in the analysis. Lactobacillus iners (62.8%) and specific BV-associated bacteria, namely, Gardnerella vaginalis (58.6%), Atopobium vaginae (40.7%), and the pathobiont Ureaplasma parvum (37.9%), were the most prevalent microbes. Hierarchical clustering analysis revealed that 42.8% of the women (62/145) had a diverse array of heterogeneously distributed bacteria typically linked to BV. Women with detectable *Lactobacillus* species, specifically Lactobacillus crispatus and Lactobacillus jensenii, and to a lesser extent *L. iners*, had very low prevalence of BV-associated bacteria. Although the cumulative burden of STIs/pathobionts was 62.8%, Chlamydia trachomatis (3.4%), Neisseria gonorrhoeae (4.8%), and Trichomonas vaginalis (4.8%) were detected at low rates. HIV infection was associated with the presence of STIs/pathobionts (*P* = 0.022) and *L. iners* (*P* = 0.003). Prevalent STIs/pathobionts were associated with having multiple partners in the past 12 months (*n* ≥ 2, *P* = 0.015), high number of lifetime sexual partners (*n* ≥ 3, *P* = 0.007), vaginal sex in the past month (*P* = 0.010), and decreasing age of women (*P* = 0.005). C. trachomatis was associated with increasing age among HIV-positive women (*P* = 0.016). The pathobiont Ureaplasma urealyticum was inversely associated with age of women in the whole cohort (*P* = 0.018). The overall prevalence of STIs/pathobionts was high and was associated with HIV infection and sexual behavior. Our study helps us to understand the epidemiological trend of STIs and pathobionts and highlights the need to understand the impact of sexual networks on STI and pathobiont transmission and prevention among women in an African setting.

**IMPORTANCE** Bacterial vaginosis (BV), whose etiology remains a matter of controversy, is a common vaginal disorder among reproductive-age women and can increase the risk for sexually transmitted infections (STIs). African women bear a disproportionately high burden of STIs and BV. Using a targeted quantitative PCR (qPCR) assay, a customized bacterial vaginosis microbial DNA qPCR array, we examined the prevalences and determinants of key cervical microbes, including BV-associated bacteria and emerging sexually transmitted pathogens (pathobionts) among women of African descent aged between 30 and 75 years. High-risk behaviors were associated with a higher prevalence of STIs/pathobionts, suggesting the need to better understand the influence of sexual networks on STI and pathobiont transmission and prevention among women. Our molecular assay is important in the surveillance of BV-associated bacteria, pathobionts, and STIs as well as diagnostic microbiology of BV. Furthermore, our research contributes to a better understanding of the epidemiology of STIs and pathobionts in Africa.

## INTRODUCTION

Common wisdom is that a preponderance of *Lactobacillus* species (specifically Lactobacillus crispatus, Lactobacillus jensenii, Lactobacillus gasseri, and Lactobacillus iners) defines a healthy cervical and vaginal (cervicovaginal) microbiota (1, 2). Lactobacilli are thought to reduce the risk of genital tract infections and syndromes by employing antipathogenic mechanisms such as production of antimicrobial compounds (e.g., lactic acid), immunomodulation, and competitive exclusion through adherence to cervicovaginal epithelial cells (2). Among the lactobacilli, L. crispatus and *L. iners* are regarded as the most and least protective, respectively (2, 3). *L. iners* can occur in healthy, transitional, and dysbiotic microbiota (2). There is evidence for ethnic variations in cervicovaginal microbiota, with *Lactobacillus*-dominated microbiota being less common in women of African descent than in non-African women (1, 4, 5). In sub-Saharan Africa, including South Africa, *L. iners*-dominated cervicovaginal microbiota are the most prevalent among microbiota with lactobacilli dominance (3, 6–8). Imbalances of vaginal microbiota can lead to bacterial vaginosis (BV), a polymicrobial disorder characterized by loss of lactobacilli concomitant with an overgrowth of coccobacilli that include Gardnerella vaginalis and other anaerobic bacteria (9).

BV can be diagnosed using Nugent scoring or Amsel’s criteria (10, 11), with the former considered the gold standard (10). Nugent scoring is based on Gram staining of vaginal smear followed by identification of bacterial morphotypes (lactobacilli and BV-associated bacteria) and scoring of microflora abnormality (0 to 3: normal microflora, 4 to 6: intermediate/mixed vaginal microflora, and 7 to 10: BV) (10). On the other hand, Amsel’s criteria rely on the presence of at least three of following four clinical findings (signs or symptoms) to define BV: high vaginal pH (>4.5), homogeneous white/gray discharge, a fishy odor following addition of 10% potassium hydroxide to vaginal fluid (positive “whiff test”), and clue cells (presence of exfoliated squamous epithelial cells with adherent coccobacilli) on wet mount (12). Amsel’s criteria are moderately reproducible and may inaccurately diagnose BV due to lack of time or expertise (10). Both Nugent scoring and Amsel’s criteria do not show detailed information of BV-associated bacteria. Molecular-based methods, such as quantitative real-time PCR targeting specific BV-associated bacteria have augmented BV diagnosis and enabled us to examine BV microflora at an unprecedented resolution (10, 11, 13, 14). Such methods are highly accurate indicators of BV since they have improved test characteristics for BV diagnostics (13–15). Therefore, these methods may be useful when used in conjunction with microscopic and clinical methods to diagnose BV and determine women at high risk for recurrent BV (16). Thus, there is a need to optimize and use molecular methods to better understand the microbiology of BV.

BV is the most frequently reported vaginal syndrome among reproductive-age women, with a global prevalence of 23 to 29% in the general population (17). Sub-Saharan African women, especially from southern Africa, have high rates of BV, with the rates differing geographically (18, 19). For example, the estimates of BV in South Africa range from 34 to 58%, with high rates (58%) reported in Cape Town and rural KwaZulu-Natal (18). Risk factors for BV include sexual behavior (19, 20), hormonal contraception, ethnicity, and use of intravaginal hygiene products (21), to mention a few.

BV has been implicated in several adverse clinical and reproductive health outcomes such as increased risk of acquiring sexually transmitted infections (STIs) (22) that range from Chlamydia trachomatis, Trichomonas vaginalis, and Neisseria gonorrhoeae to HIV infection (22, 23). Positive associations of BV and BV-associated bacteria with HIV infection have been documented (24–26). BV-associated bacteria are known to increase the HIV-1 viral replication and shedding in HIV target cells (27). Furthermore, BV-associated bacteria such as *G. vaginalis* and cervicovaginal microbiota with paucity of lactobacilli have been associated with disruption of epithelial barriers (28) and human papillomavirus (HPV) infection (29–31). Infection with persistent high-risk HPV (HR-HPV) types causes cervical cancer (32), the leading cancer affecting women aged 15 to 44 years in southern Africa (33). Both HIV and HPV infections are highly prevalent in South Africa (33–36). Hence, considering the high burden of BV, its recalcitrance to antibiotic therapy (8), and its association with STIs, accurate approaches for diagnosis and effective treatment are essential. Knowledge of epidemiological trends of BV and STIs may inform policy decisions concerning their prevention and control strategies.

Owing to the high burden of BV among women in Africa, there is a need for more epidemiological data on the prevalences of BV-associated microbes, especially using targeted PCR assays, in order to determine the true prevalences of these microbes and assess their utility as diagnostic markers. Until now, the relationship of BV-associated bacteria and STIs with HIV remains poorly understood, with some authors finding no statistically significant associations between BV and individual STIs detected through Nugent scoring and quantitative PCR (qPCR), respectively (37). In a recent study (38), we employed a multiplex PCR-based STD direct flow chip assay to investigate the prevalence of 12 STIs and emerging sexually transmitted pathogens (pathobionts, commensal bacteria with pathogenic potential) among rural women (aged ≥30 years) with a high burden of HR-HPV (32.2%) and HIV-1 (38.5%) recruited from a rural community-based clinic in Eastern Cape (South Africa). These microbes included C. trachomatis (serovars L1 to L3 and serotypes A to K), herpes simplex virus (types I and II), T. vaginalis, *N. gonorrhoeae*, Treponema pallidum, Haemophilus ducreyi, Mycoplasma hominis, Mycoplasma genitalium, and ureaplasmas (Ureaplasma urealyticum or Ureaplasma parvum). Whereas the study found the overall prevalences of STIs (22.9%) and pathobionts to be high (83.9%), it was only limited to 12 microbes and underexplored BV-associated microbes. We therefore aimed to use a customized molecular method, bacterial vaginosis microbial DNA qPCR array, to screen for a wide range of microbes (*n* = 38), mostly BV-associated bacteria and pathobionts in cervical samples from our previously highlighted study. In addition, participant characteristics associated with STI as well as STIs and pathobionts associated with HIV infection were investigated.

## RESULTS

### Demographic characteristics of study participants.

The final analysis was done on 145 participants (89.5%, 145/162). A total of 17 (10.5%) samples were excluded from the analysis for the following reasons: (i) no bacterium and protozoon included in the assay was detected and/or (ii) any control included in the assay had failed. The description of the 145 participants finally included in the study is summarized in Table 1. While the age of the women ranged from 30 to 75 years, their median age (43 years) was that of perimenopausal age. More than a third (37.9%) of the women were HIV-positive. Of these, 99.0% were on antiretroviral drugs. A small proportion (11.7%) of the women had abnormal cervical cytology (atypical squamous cells of undetermined significance [ASCUS]: 76.5% [13/17], low-grade squamous intraepithelial lesion [LSIL]: 17.6% [3/17], high-grade squamous intraepithelial lesion [HSIL]: 5.9% [1/17]).

**TABLE 1:** Description of study participants^*a*^

Variable	All participants (*N* = 145)
Age (yrs)	43 [37–52]^*b*^
Menopausal age (yrs) (*n*/*N*)	
30–49	66.2 (96/145)
50–75	33.8 (49/145)
HIV status (% [*n*/*N*])	
Negative	62.1 (90/145)
Positive	37.9 (55/145)
Cervical cytology (% [*n*/*N*])	
Normal	88.3 (128/145)
Abnormal	11.7 (17/145)
Sexual behavior	
Age at first sex (yrs)	18 [16–20]
Lifetime no. of sexual partners	3 [2–4]
No. of sexual partners past in the last 12 mo	1 [1–1]
Contraceptive use (with current partner)? (*n*/*N*)	
No	56.6 (82/145)
Yes	42.8 (62/145)
If above is yes, method of contraception (with current partner) (*n*/*N*)	
Condoms	46.8 (29/62)
Injectables/birth control pills	48.4 (30/62)
Tubal ligation/intrauterine device	4.8 (3/62)
Use of condom (last time) (*n*/*N*)	
No	56.5 (82/145)
Yes	41.4% (60/145)
Ever been pregnant? (*n*/*N*)	
No	4.1 (6/145)
Yes	95.9 (139/145)
Any miscarriages, abortions/ectopic pregnancy? (*n*/*N*)	
No	27.6 (40/145)
Yes	72.4 (105/145)
Ever experienced vaginal discharge? (*n*/*N*)	
No	50.3 (73/145)
Yes	49.7 (72/145)

a HIV, human immunodeficiency virus.

b Continuous variables are expressed as medians with interquartile ranges (IQRs; at the 25th and 75th percentiles).

The distribution of the above-described baseline characteristics according to the age group of the women is tabulated in the supplementary information (see Table S1 at https://doi.org/10.6084/m9.figshare.19714483). In addition, this table statistically compares the variables of women aged 30 to 39 years with those aged 40 to 49 and over 50 years. Overall, a significantly higher proportion of women aged 30 to 39 years had high-risk behaviors such as multiple sexual partners in the past 1 year, lifetime sexual partners, higher frequency of vaginal sex in the last 1 month, and so on, compared to their counterparts.

### Prevalence of the cervical microbes.

**FIG 1:**
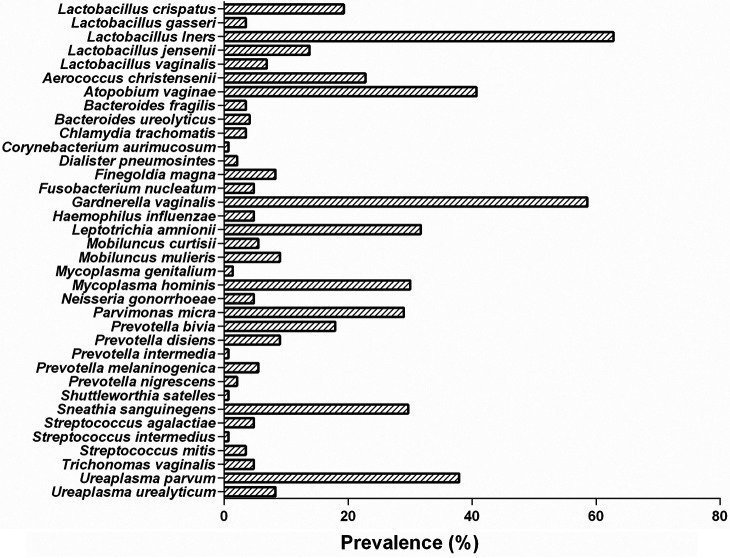
Prevalence of cervical microbes among South African women attending a community-based clinic.

Of the 38 microbes that we sought to identify, 36 were detected. Clostridium sordellii and Finegoldia magna were not detected. The prevalences of the cervical microbes detected in our cohort are shown in Fig. 1. The most frequently detected microbes were *L. iners* (62.8%, 91/145) and the common BV-associated bacteria, *G. vaginalis* (58.6%, 85/145), Atopobium vaginae (40.7%, 59/145), *U. parvum* (37.9%, 55/145), Leptotrichia amnionii (31.7%, 46/145), and Sneathia sanguinegens (29.7%, 43/145). The least frequently detected microbes, with a prevalence of ≤5%, included Corynebacterium aurimucosum, Prevotella intermedia, Shuttleworthia satelles, Streptococcus intermedius, etc.

Because of their notable importance in human reproductive health and disease, it is worth mentioning the detection rates of any *Lactobacillus* spp. as well as other *Lactobacillus* spp. besides *L. iners*. About three-quarters of the women (74.5%) had any detectable *Lactobacillus* spp., with nearly a third (31.5%) of them having multiple species (2 species: 25 women, 3 species: 7 women, and 4 species: 2 women). The prevalences of the other *Lactobacillus* spp. besides *L. iners* were as follows: L. crispatus (19.3%, 28/145), L. jensenii (13.8%, 20/145), *L. vaginalis* (6.9%, 10/145), and L. gasseri (3.4%, 5/145).

The cumulative prevalence of STIs and pathobionts was 62.8% (91/145). The prevalences of C. trachomatis, *N. gonorrhoeae*, and T. vaginalis were 3.4% (5/145), 4.8% (7/145), and 4.8% (7/145), respectively. All pathobionts, including *U. parvum* (37.9%, 55/145), the most prevalent, were detected at considerably varied rates (Mycoplasma hominis: 29.0% [42/145], *U. urealyticum*: 8.3% [12/145], and M. genitalium: 1.4% [2/145]).

### Hierarchical clustering of the detected cervical microbes.

**FIG 2:**
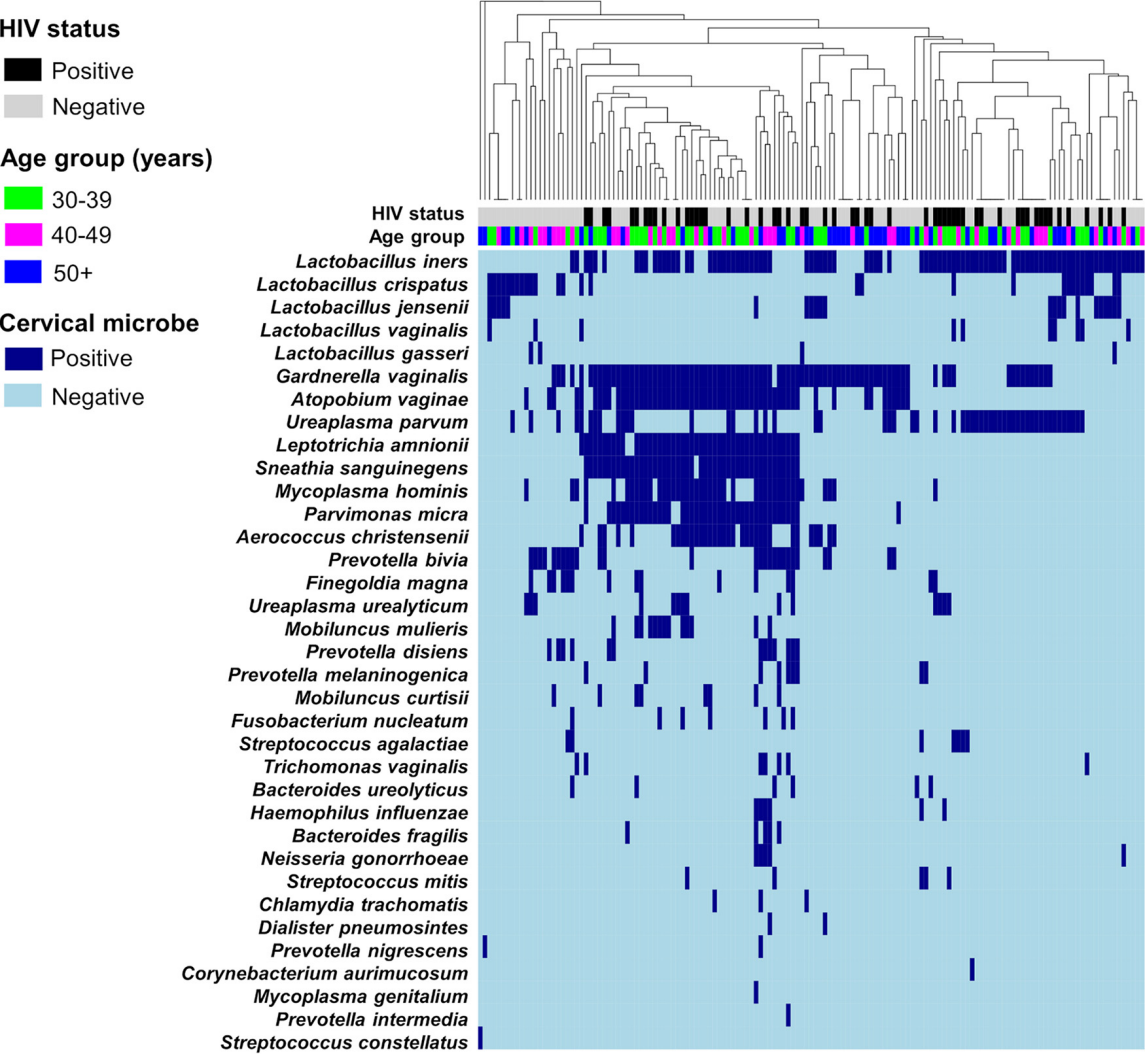
Heatmap showing microbes detected in cervical samples of 145 South African women. Detectable cervical microbes are presented on the *y* axis. Except for L. crispatus, L. jensenii, *L. vaginalis*, and L. gasseri, all the microbes are ranked according to decreasing prevalence. Each participant is presented on the *x* axis. The dendrogram on top of the binary heatmap is based on average linkage hierarchical clustering of the Jaccard dissimilarity index.

We observed that the samples clustered according to the presence of cervical microbes (Fig. 2), although the algorithm (gap statistic) did not converge toward an optimal solution in 10 iterations. The heatmap also shows the distribution of the detected microbes across the women. Of the 145 women, 42.8% (62/145) had a diverse array of heterogeneously distributed bacteria (e.g., *G. vaginalis*, *A. vaginae*, *L. amnionii*, and *S. sanguinegens*). Women with detectable *Lactobacillus* spp., specifically L. crispatus and L. jensenii, and to a lesser extent *L. iners*, had very low prevalence of BV-associated bacteria. Interestingly, 42.9% (39/91) of women with detectable *U. parvum* had detectable *L. iners* but without a diverse group of BV-associated bacteria.

### Association of *Lactobacillus* species with HIV status and age group.

Owing to the documented inverse correlation between a woman’s reproductive stage and the levels of *Lactobacillus* spp. (39), as well as the roles of these *Lactobacillus* spp. in HIV infection (3, 40), we investigated whether there was any association of *Lactobacillus* spp. with the women’s age group and HIV infection in our cohort. The associations of *Lactobacillus* spp. with the age group of women are summarized in Table 2. About three-quarters (74.5%, 108/145) of the women in our cohort had any detectable *Lactobacillus* spp. We noted that the prevalences of any detectable *Lactobacillus* spp. (i.e., genus *Lactobacillus*) and *L. iners* significantly decreased with increasing age group (*P* = 0.019 and *P* = 0.005, respectively). None of the other individual *Lactobacillus* spp. varied by the age group of the women.

**TABLE 2:** Prevalence of *Lactobacillus* species according to age

Variable	N	Data for age group:			
		30–39 yrs (%) (*n*/*N*)	40–49 yrs (%) (*n*/*N*)	≥50 yrs (%) (*n*/*N*)	*P* value
Any *Lactobacillus* sp.					
Negative	37	13.0 (7/54)	28.6 (12/42)	36.7 (18/49)	Ref^*a*^
Positive	108	87.0 (47/54)	71.4 (30/42)	63.3 (31/49)	0.019
Lactobacillus crispatus					
Negative	117	83.3 (45/54)	83.3 (35/42)	75.5 (37/49)	Ref
Positive	28	16.7 (9/54)	16.7 (7/42)	24.5 (12/49)	0.529
Lactobacillus gasseri					
Negative	140	98.1 (53/54)	92.9 (39/42)	98.0 (48/49)	Ref
Positive	5	1.9 (1/54)	7.1 (3/42)	2.0 (1/49)	0.441
Lactobacillus iners					
Negative	54	20.4 (11/54)	45.2 (19/42)	49.0 (24/49)	Ref
Positive	91	79.6 (43/54)	54.8 (23/42)	51.0 (25/49)	0.005
Lactobacillus jensenii					
Negative	125	85.2 (46/54)	90.5 (38/42)	83.7 (41/49)	Ref
Positive	20	14.8 (8/54)	9.5 (4/42)	16.3 (8/49)	0.626
Lactobacillus vaginalis					
Negative	135	87.0 (47/54)	97.6 (41/42)	95.9 (47/49)	Ref
Positive	10	13.0 (7/54)	2.3 (1/42)	4.1 (2/49)	0.114

a Ref, reference.

When we stratified our cohort by HIV status, we found that there was no difference in the prevalence of any *Lactobacillus* spp. between women with and without HIV infection (odds ratio [OR]: 1.9 [95% confidence interval (CI): 0.8 to 4.4], *P* = 0.113; Table 3). However, HIV-positive women were less likely to have detectable L. crispatus than HIV-negative women (OR: 0.3 [95% CI: 0.1 to 0.8], *P* = 0.015). The prevalence of *L. iners* was significantly higher in HIV-positive women than in HIV-negative women (OR: 3.1 [95% CI: 1.5 to 6.7], *P* = 0.003).

**TABLE 3:** Association of *Lactobacillus* species with HIV infection^*a*^

Variable	*N*	HIV-positive (%) (*n*/*N*)	HIV-negative (%) (*n*/*N*)	OR (95% CI)	*P* value
Any *Lactobacillus* sp.					
Negative	37	18.2 (10/55)	30.0 (27/90)		Ref
Positive	108	81.8 (45/55)	70.0 (63/90)	1.929 (0.849–4.381)	0.113
Lactobacillus crispatus					
Negative	117	90.9 (50/55)	74.4 (67/90)		Ref
Positive	28	9.1 (5/55)	25.6 (23/90)	0.291 (0.104–0.820)	0.015
Lactobacillus gasseri					
Negative	140	98.2 (54/55)	95.6 (86/90)		Ref
Positive	5	1.8 (1/55)	4.4 (4/90)	0.398 (0.043–3.659)	0.650
Lactobacillus iners					
Negative	54	21.8 (12/55)	46.7 (42/90)		Ref
Positive	91	78.2 (43/55)	53.3 (48/90)	3.135 (1.463–6.720)	0.003
Lactobacillus jensenii					
Negative	125	89.1 (49/55)	84.4 (76/90)		Ref
Positive	20	10.9 (6/55)	15.6 (14/90)	0.665 (0.239–1.847)	0.431
Lactobacillus vaginalis					
Negative	135	94.5 (52/55)	92.2 (83/90)		Ref
Positive	10	5.5 (3/55)	7.8 (7/90)	0.684 (0.169–2.765)	0.592

a HIV, human immunodeficiency virus; OR, odds ratio; CI, confidence interval; Ref, reference.

### Associations of STIs and pathobionts with HIV status and age group.

Next, we assessed the associations of STIs and pathobionts with HIV status. A great proportion of women (62.8%, 91/145) were positive for any STI and/or pathobiont. The prevalence of any STI and/or pathobiont was significantly higher in HIV-positive women than in HIV-negative women (OR: 2.3 [95% CI: 1.1 to 4.9], *P* = 0.022; Table 4). Neither the detection rates of the individual STIs nor those of the pathobionts were associated with HIV infection.

**TABLE 4:** Association of STIs and pathobionts with HIV status^*a*^

Variable	*N*	HIV-positive (%) (*n*/*N*)	HIV-negative (%) (*n*/*N*)	OR (95% CI)	*P* value
Any STI and/or pathobiont					
Negative	54	25.5 (14/55)	44.4 (40/90)		Ref
Positive	91	74.5 (41/55)	55.6 (50/90)	2.343 (1.123–4.889)	0.022
Chlamydia trachomatis					
Negative	140	92.7 (51/55)	98.9 (89/90)		Ref
Positive	5	7.3 (4/55)	1.0 (1/90)	6.980 (0.759–64.190)	0.069
*Neisseria gonorrhoeae*					
Negative	138	94.6 (52/55)	95.6 (86/90)		Ref
Positive	7	5.5 (3/55)	4.4 (4/90)	1.240 (0.267–5.777)	1.000
Mycoplasma genitalium					
Negative	143	98.2 (54/55)	98.9 (89/90)		Ref
Positive	2	1.8 (1/55)	1.0 (1/90)	1.648 (0.101–26.920)	1.000
Mycoplasma hominis					
Negative	103	63.6 (35/55)	75.6 (68/90)		Ref
Positive	42	36.4 (20/55)	24.4 (22/90)	1.766 (0.851–3.666)	0.125
Trichomonas vaginalis					
Negative	138	90.9 (50/55)	97.8 (88/90)		Ref
Positive	7	9.1 (5/55)	2.2 (2/90)	4.400 (0.823–23.530)	0.105
Ureaplasma urealyticum					
Negative	133	89.1 (49/55)	93.3 (84/90)		Ref
Positive	12	10.9 (6/55)	6.7 (6/90)	1.143 (0.384–3.405)	0.810
Ureaplasma parvum					
Negative	90	60.0 (33/55)	63.3 (57/90)		Ref
Positive	55	40.0 (22/55)	36.7 (33/90)	1.152 (0.578–2.294)	0.688

a STI, sexually transmitted infection; HIV, human immunodeficiency virus; OR, odds ratio; CI, confidence interval; Ref, reference.

We further investigated demographic, sexual behavioral, and clinic characteristics of the participants associated with STI/pathobiont. The odds of STI/pathobiont positivity were significantly higher in women having 2 or more sexual partners in the past 1 year (OR: 13.3 [95% CI: 1.5 to 115.9], *P* = 0.005), 3 or more lifetime sexual partners (OR: 3.7 [95% CI: 1.4 to 10.0], *P* = 0.007), and frequency of vaginal sex (once to thrice) in the last month (OR: 2.8 [95% CI: 1.3 to 6.3], *P* = 0.010; Table 5).

**TABLE 5:** Association of STI and pathobiont baseline characteristics of the participants^*a*^

Variables	STI and/or pathobiont prevalence (%) (*n*/*N*)	OR (95% CI)	*P* value
Age at first sexual intercourse			
<16	59.1 (13/22)		Ref
16–18	68.8 (53/77)	1.529 (0.575–4.063)	0.393
>18	54.3 (25/46)	0.824 (0.294–2.307)	0.713
No. of sexual partners in past in the 12 mo			
0	42.9 (15/35)		Ref
1	66.7 (66/99)	2.667 (1.211–5.872)	0.013
≥2	90.9 (10/11)	13.330 (1.534–115.900)	0.005
Lifetime sexual partners			
1	40.9 (9/22)		Ref
2	56.8 (25/44)	1.901 (0.673–5.370)	0.223
≥3	72.2 (57/79)	3.742 (1.401–9.994)	0.007
Frequency of vaginal sex in the last mo			
0	52.2 (36/69)		Ref
1–3	75.5 (37/49)	2.826 (1.264–6.319)	0.010
≥4	65.4 (17/26)	1.731 (0.679–4.415)	0.248
Use of condom (last time)			
No	68.3 (56/82)		Ref
Yes	56.7 (34/60)	0.607 (0.304–1.212)	0.156
Method of contraception last time			
No method/withdrawal	66.0 (33/50)		Ref
Condoms	50.0 (25/50)	0.515 (0.230–1.154)	0.105
Injectables/birth control pills	74.4 (29/39)	1.494 (0.591–3.775)	0.395
Tubal ligation	75.0 (3/4)	1.545 (0.149–16.010)	1.000
Contraceptive use (with current partner)			
No	58.5 (48/82)		Ref
Yes	69.4 (43/62)	1.603 (0.799–3.216)	0.183
Method of contraception (with current partner)			
Condoms	58.6 (17/29)		Ref
Injectables/birth control pills	76.7 (23/30)	2.319 (0.754–7.134)	0.138
Tubal ligation/intrauterine device	66.7 (2/3)	1.412 (0.115–17.410)	1.000
History of vaginal discharge			
No	60.3 (44/73)		Ref
Yes	65.3 (47/72)	1.239 (0.631–2.433)	0.533
Frequency of vaginal discharge			
No discharge	60.3 (44/73)		Ref
Current/last wk	59.1 (13/22)	0.952 (0.3606–2.514)	0.921
>1 wk and <1 mo	55.6 (10/18)	0.824 (0.291–2.334)	0.715
≥6 mo	74.2 (23/31)	1.895 (0.747–4.809)	0.175

a STI, sexually transmitted infection; OR, odds ratio; CI, confidence interval; Ref, reference.

**FIG 3:**
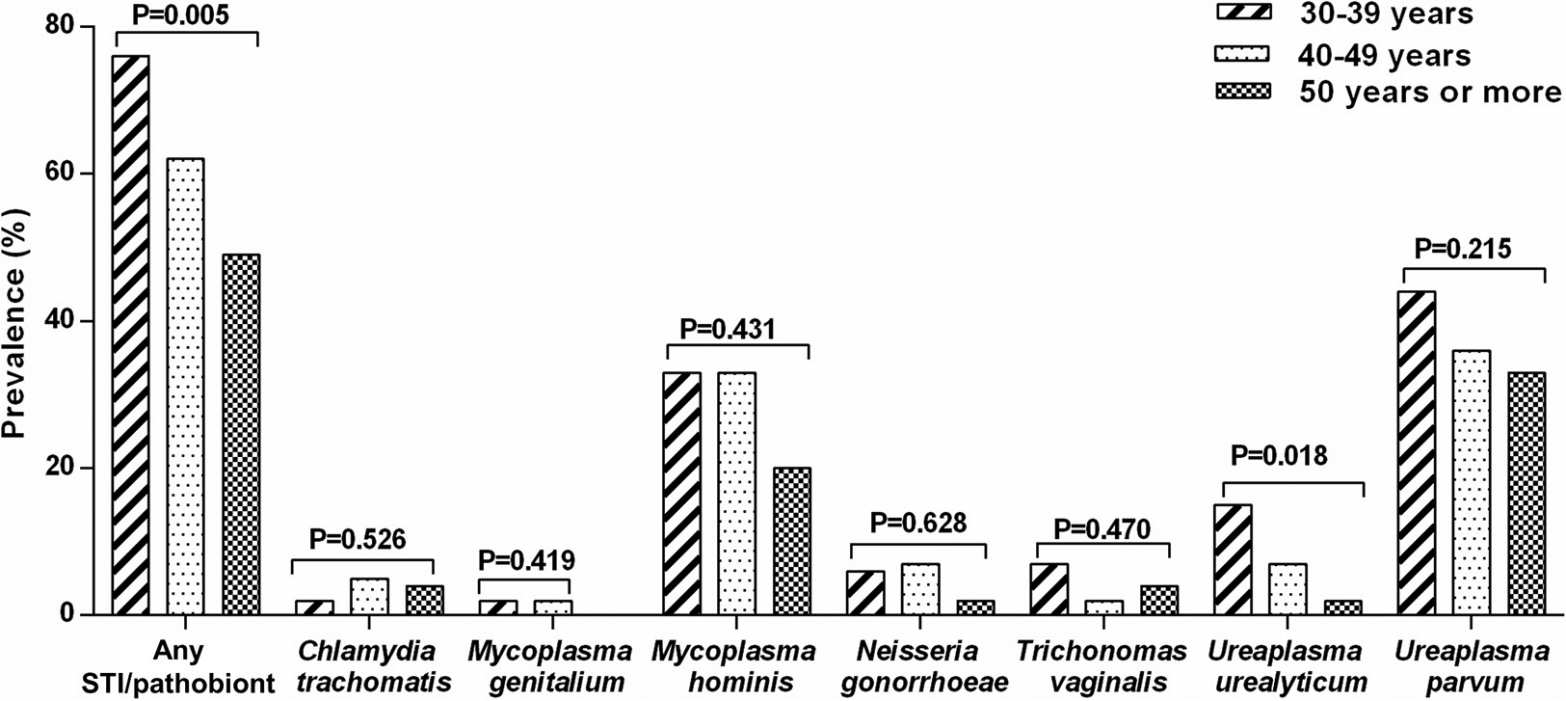
Cumulative prevalence of STI/pathobiont, individual STIs, and individual pathobionts according to age category of the women in the whole cohort.

Finally, we also attempted to evaluate the distribution of the STIs and pathobionts according to the age group of women. We noted that the cumulative prevalence of STIs and pathobionts significantly decreased as the age of women increased (*P* = 0.005; Fig. 3). An analogous observation was seen regarding *U. urealyticum* (*P* = 0.018; Fig. 3). There were no significant associations between the other microorganisms, including T. vaginalis, and age category. All these observations remained unchanged among women with normal cytology (any STI/pathobiont: *P* = 0.015, *U. urealyticum*: *P* = 0.014; see Fig. S1 at https://doi.org/10.6084/m9.figshare.19714483). A trend of an inverse relationship between the prevalence of *M. hominis* and age was observed among women with normal cytology (*P* = 0.085; see Fig. S1 at the URL mentioned above). We did not explore the distribution of the STIs and pathobionts among women with abnormal cytology because of the small number of subjects in this subgroup (*n* = 17, 58.9% of which were positive for any STI/pathobiont).

**FIG 4:**
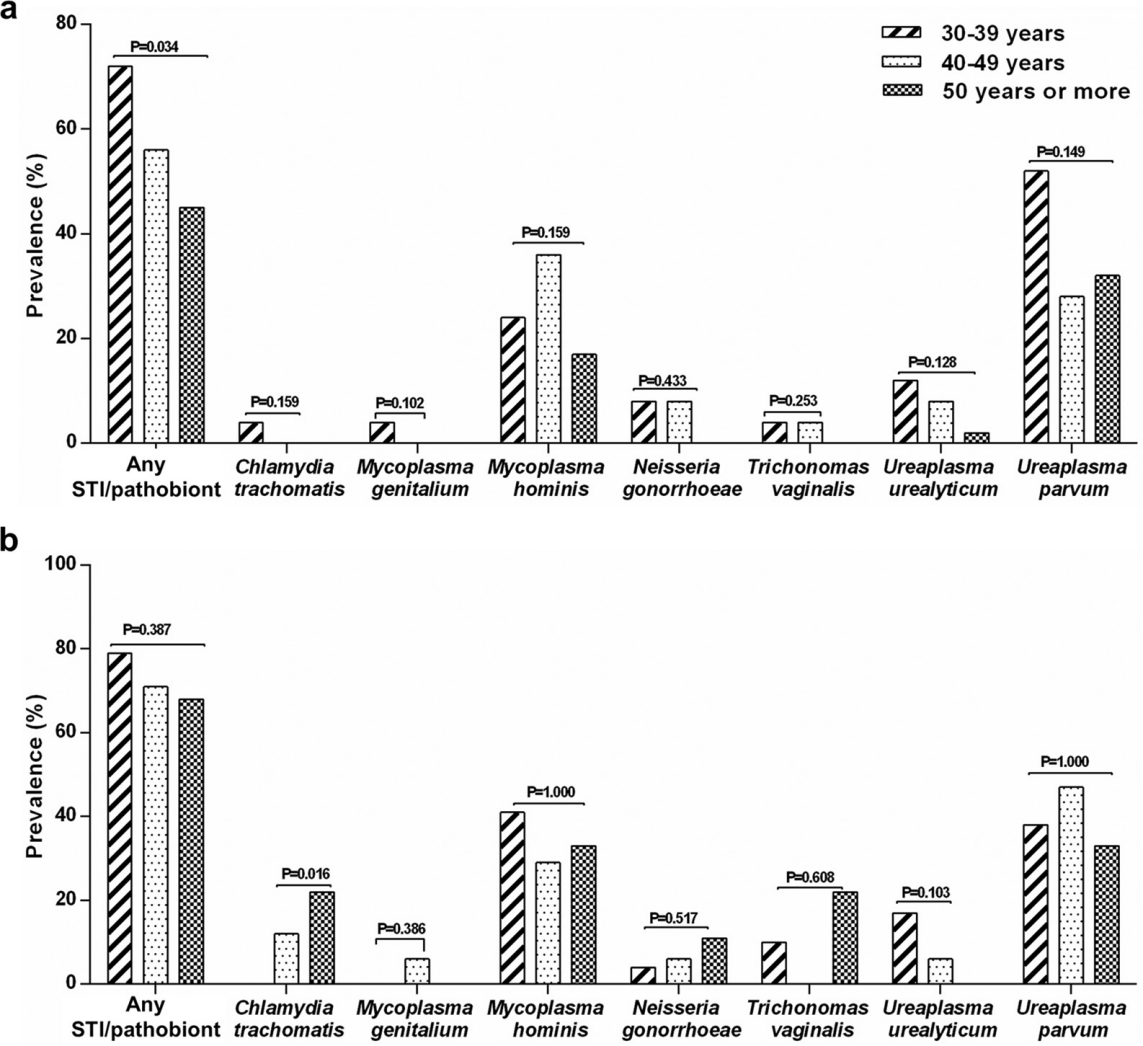
Cumulative prevalence of STI/pathobiont, individual STIs, and individual pathobionts according to HIV status and age category of women. (a and b) Prevalence of STIs and pathobionts in HIV-negative women (a) and HIV-positive women (b).

Further analyses showed that the cumulative prevalence of STIs and pathobionts decreased with increasing age among HIV-negative women (*P* = 0.034; Fig. 4a), but not among HIV-positive women (*P* = 0.387; Fig. 4b). The prevalence of C. trachomatis significantly increased with increasing age, but only in HIV-positive women (*P* = 0.016; Fig. 4b).

## DISCUSSION

With the growing consensus recognizing the adverse sequalae associated with BV, there is need for more data on the prevalence of cervicovaginal BV-associated microbes, especially in regions burdened with STIs. These data may have applications in BV diagnostics, routine surveillance, and public health decision-making. Our study is the first of its kind to employ a customized molecular technique to examine the prevalences and determinants of selected cervical microbes among women with and without HIV infection in rural Eastern Cape, South Africa. The pattern of the most commonly detected cervical bacteria in our study (e.g., *L. iners* and the BV-associated bacteria *G. vaginalis*, *A. vaginae*, *L. amnionii*, and *S. sanguinegens*) is congruent with previous reports (13, 31, 41), including those that have used qPCR assays (14, 15, 42). A small cohort study on a population of predominately African-American women at high risk for STIs, noted that the most prevalent vaginal species as detected by qPCR was *L. iners* (42).

The aforesaid BV-associated bacteria are known to occur in high prevalences and abundances in women with BV (8, 13, 14, 37, 41, 42). A study that used a multiplex real-time PCR assay to examine BV among 151 women (mostly Dutch Caucasians) aged 18 to 62 years found that *G. vaginalis* and *A. vaginae* were remarkably more common in women with BV than in women without BV (*G. vaginalis*: 96% versus 27%, *A. vaginae*: 87% versus 6%, respectively) (14). This observation was echoed in a subsequent study of 37 women (median age: 26 years), mostly African American, clinically diagnosed as having either normal, intermediate, or BV microfloras. By utilizing targeted qPCR assays, this particular study noted that the prevalences of BV-associated bacteria, including *G. vaginalis*, increased with severity of BV (42). Among the BV-associated bacteria, *G. vaginalis* is regarded as the most virulent (43). It has the capacity to displace the protective lactobacilli from the vaginal epithelial cells (44) and is probably partly responsible for BV treatment failure (8). It can also form an adherent robust biofilm on the vaginal epithelium, thereby allowing other opportunistic pathogens to colonize the genital econiche (43). It is believed that such changes may lead to the establishment of BV (44). Omics approaches for studying microbiota have revealed that BV and cervicovaginal microbiota with *G. vaginalis* dominance have unique functional signatures (28, 45), which may compromise cervicovaginal epithelial integrity barrier to infections (28). It is no wonder that specific detectable BV-associated bacteria (e.g., *S. sanguinegens*) are associated with genital inflammation (40), increased HIV risk (25, 40), and HR-HPV infection (31). We did not detect *C. sordellii* and *F. magna*, which are extremely unusual bacteria in the female genital tract, including those of African women (4).

The cumulative prevalence of *Lactobacillus* spp. (75%) in our study was lower than in other culture-independent studies of African cohorts (85 to 100%) (3, 6, 31, 46). Perhaps this could be due to disparities in study population and methodology, including performances of qPCR versus 16S rRNA gene amplicon sequencing. Similar to culture-independent studies of South African cohorts (6, 31, 46), we further found that *L. iners* was the most prevalent *Lactobacillus* spp. This observation is in good agreement with studies that have employed qPCR assays to study cervicovaginal bacteria (14, 15, 37, 42). Culture-based studies have reported a lower prevalence of *L. iners* than molecular-based studies. For example, a small-cohort culture-based study on reproductive-age South African women without HIV infection observed more than 2-fold lower prevalence of *L. iners* than in our study (27% versus 63%) (47). Of course, this difference emanates mainly from the higher sensitivity of DNA technologies and their inherent ability to detect DNA from both viable and nonviable cells (48), difficulty in culturing *L. iners* (47), and differences in the study population (including HIV status and sample size). The observed low prevalence of the other common cervicovaginal lactobacilli is not surprising since it mirrors previous reports using culture (47) and targeted PCR assays (14, 42) and high-throughput amplicon sequencing of the hypervariable region of 16S rRNA gene (6, 31, 46). Women of African descent are less frequently colonized by these *Lactobacillus* spp. than Caucasian women and Asian women (13). Instead, most of them have microbiota characterized by high diversity contributed by an array of non-*Lactobacillus* spp. (1, 3, 5). The composition of such microbiota could be governed by host ethnicity or genetics (1, 5, 46) and sexual behavior (7, 20, 49) among other factors.

We noted that older (perimenopausal, menopausal, and postmenopausal) women in our study had a significantly lower prevalence of any detectable *Lactobacillus* spp. and *L. iners* than that of younger (premenopausal) women. This qualitative observation is consonant with quantitative analyses of lactobacilli on premenopausal versus postmenopausal women (26, 39, 50), further confirming that the composition of cervicovaginal microbiota is impacted by reproductive aging (26). There is a general opinion that the mechanism of estrogen action on glycogen synthesis, which varies by a woman’s reproductive stage, is responsible for this. As a woman ages, the levels of glycogen diminish in tandem with estrogen levels. *Lactobacillus* spp. depend on cell-free glycogen, produced by vaginal epithelial cells, as their main carbon source (50). So, reduction of estrogen levels causes thinning of the glycogen vaginal epithelial layer, thereby resulting in low levels of lactobacilli (39). Also, cell-free glycogen levels have been negatively correlated with vaginal pH (51), which seems to be higher in postmenopausal women than in premenopausal women. High vaginal pH may create a favorable growth environment for opportunistic pathogens, potentially increasing the risk of acquiring STIs. Hence, estrogen replacement therapy may be used to restore *Lactobacillus* colonization in women with deficient lactobacilli (e.g., postmenopausal women), thereby maintaining cervicovaginal health and protecting such against genital tract infections (50).

We further found that detectable L. crispatus and *L. iners* were negatively and positively associated with prevalent HIV infection, respectively. While this resonates with previous cross-sectional studies of HIV-infected and HIV-uninfected women, there are still mixed findings on the relationship of *L. iners* with prevalent HIV (3, 52). Despite this, a review of literature on lactobacilli as biomarkers for vaginal health suggests that cervicovaginal microbiota colonized with L. crispatus are more protective against HIV infection than those colonized with *L. iners* (2). One likely explanation for this is the fact that L. crispatus produces considerably higher levels of d-lactic acid than *L. iners* does. It has been demonstrated that relatively high levels of d-lactic acid is a potent antimicrobial product that reduces permissiveness of cervicovaginal mucus to HIV infection (2). We also posit that the positive association of *L. iners* with prevalent HIV infection in our cohort could be due to the cooccurrence of BV-associated bacteria, some of which have been associated with HIV acquisition (25, 40). It is thought that *L. iners* is a transitional species that can facilitate the shift of cervicovaginal microbiota to healthy or dysbiotic (including BV) state (2).

Since there is paucity of data on STI and pathobiont burden among South African women with and without HIV infection in rural Eastern Cape, we investigated the prevalences and risk factors of these microbes. The overall prevalence of both STIs and pathobionts in our present study is at least 2.5-fold higher than we previously reported using the STD direct flow chip assay (38), thus reflecting differences in sensitivity of the assays (including the cycle threshold [*C_T_*] cutoff value that we chose) and maybe sample selection. Compared to older HIV-negative women, younger HIV-negative women had a higher prevalence of STIs/pathobionts. The most plausible explanation for this is either a higher prevalence of STIs/pathobionts among sexual partners or high-risk behaviors among younger women (53). The latter was particularly apparent in our study (see Table S1 at https://doi.org/10.6084/m9.figshare.19714483). Hence, there is a need to understand the importance of the sexual network in the transmission of STIs/pathobionts. We further observed a positive association between STIs/pathobionts and HIV infection. An intimate association of HIV with STIs (54, 55) and specific pathobionts (25, 40) has been published and is believed to be contributed by persistent high-risk sexual behaviors (54, 55), such as increased likelihood of engaging in condomless anal and vaginal intercourse among HIV-positive individuals unaware of their (HIV) status (56). This in turn increases the risk of STI/HIV transmission in the community. These findings reiterate the need to intensify STI/HIV testing and counselling services, treatment of STI/HIV, and advocating for behavioral change (e.g., correct and consistent condom use).

We noted that the prevalences of the individual STIs were low (each <5%) yet both consistent (38) and inconsistent (53, 55) with previous reports on South African cohorts. This consistency is certainly because a large proportion of our present cohort was part of our previous study (38) of the STD direct flow chip assay. This low prevalence could be attributed to the fact that the women were older (perhaps with low-risk behaviors) and maybe receiving STI treatment. The increase in prevalent Chlamydia infection with increasing age among HIV-positive women in our study could be a result of either (i) immunobiological factors (i.e., low paucity of *Lactobacillus* spp., less acidic vaginal pH, and/or altered immune responses, including reduced levels of interferon gamma [IFN-γ] and interleukin-17 [IL-17] [57], as women age) or (ii) sexual behavior of the partner (since women aged ≥40 years reported a smaller number of new sexual partners). The latter explanation suggests that biomedical and structural (sociobehavioral) interventions targeting both sexual partners might have an impact on the reduction of the burden of STI.

The distribution pattern of the pathobionts in our present study was comparable to the results of the STD direct flow chip assay (38), with ureaplasmas (largely contributed by *U. parvum*) and *M. hominis* being the most prevalent. In agreement with published data (58), we found that the detectability of *U. urealyticum* was associated with younger age of the women. This may be directly linked to differences in sexual behavior. While expert opinions on the relevance of cervicovaginal pathobionts on reproductive health remain unclear and divided, a few observational studies have suggested that these pathobionts could be markers or symbionts of BV microflora (1, 59) and opportunistic pathogens associated with Chlamydia infection (58) and clinical AIDS stage (60). Thus, further studies are needed to investigate the roles of these pathobionts in urogenital health in order to determine if their routine screening and treatment are warranted.

The present study has limitations that include the reliance on self-reported sexual histories and practices and vaginal discharge histories, which might have resulted in bias during collection of participant information and analysis. Second, we lacked information on BV status and other factors that could have been possible confounders in our study. Third, the customized bacterial vaginosis microbial DNA qPCR array that we used allowed us to detect only a limited number of cervical microbes. Moreover, we used an approach of the assay that does not provide the microbial loads, which could have been useful in examining correlations between cervical microbes and could be a better predictor of disease than qualitative data. We also believe that the *C_T_* cutoff value for positivity used in our assay could have limited the detection of some cervical microbes in some of the women. Lastly, our participants were recruited from a community-based clinic of the rural Eastern Cape Province in South Africa. Therefore, this may somewhat restrict the generalizability of our study results. Notwithstanding these limitations, our study highlights the potential of a customized bacterial vaginosis microbial DNA qPCR array for analysis of selected key BV-associated microbes, including pathobionts and STIs in the genital econiche.

### Conclusion.

Using a customized bacterial vaginosis microbial DNA qPCR array, we identified the microbial taxonomic profiles of cervical samples from women (aged ≥30 years) with and without HIV infection attending a rural community-based clinic in South Africa. While the prevalence of individual STIs (C. trachomatis, *N. gonorrhoeae*, and T. vaginalis) was low (each <5%), we noted that the cumulative burden of STIs and emerging sexually transmitted pathogens (M. genitalium, *M. hominis*, *U. parvum*, and *U. urealyticum* pathobionts) was high (over 60%) and that this was strongly associated with HIV infection and sexual behavior. This demonstrates the need to understand the epidemiological trend of STIs and pathobionts as well as the patterns of sexual networks and their impact on STI and pathobiont transmission, prevention, and control among different demographic groups.

## MATERIALS AND METHODS

### Study design, study population, and sample collection.

A total of 162 women were selected from a cross-sectional study conducted between September 2017 and June 2018. These were part of the study participants recruited from a community-based clinic of the rural Eastern Cape Province in South Africa as previously described (35, 38). A majority (87.7%) of these women had previously been included in our qualitative molecular diagnostic study (38) that utilized the STD direct flow chip assay. All the 162 participants were either attending cervical cancer screening or visiting the clinic for any other reasons. Inclusion criteria included women aged ≥30 years. The exclusion criteria included women <30 years old. Participants who had undergone hysterectomy were not eligible to participate in the parent and present studies. Women were requested to test for HIV if they were not aware of their HIV status or if their HIV status was not documented on their health card. Women received counselling prior to and after testing for HIV using a rapid test (Alere Determine HIV-1/2 Ag/Ab Combo; Alere, Waltham, MA, USA). Since the parent study was designed for HPV and cervical cancer screening, cervical samples were collected from women who voluntarily agreed to participate in the study using a cytobrush, stored in a Digene specimen transport medium (Qiagen, Germantown, MD, USA) and kept at −80°C until DNA extraction. Cytobrush samples are known to be more suitable for sampling the transformation zone of the cervix—since the cytobrush has a greater exfoliative ability than swab samples. As a result, they can provide a reliable and robust sampling tool for cervicovaginal microbiota evaluation (61).

### DNA extraction.

DNA was extracted from each cervical sample (400 μL) using the MagNA Pure compact nucleic acid isolation kit (Roche Diagnostics, Mannheim, Germany) on an automated MagNA Pure compact machine, in accordance with the manufacturer’s recommendations. DNA was eluted in 100 μL elution buffer followed by quantification of the DNA using a NanoDrop spectrophotometer ND-1000 (Inqaba Biotec, Pretoria, South Africa). The purified DNA was stored at −20°C until microbial analysis using the bacterial vaginosis microbial DNA qPCR array.

### Identification of microbes using a customized bacterial vaginosis microbial DNA qPCR array.

To screen the microbes in the cervical samples, we used a customized bacterial vaginosis microbial DNA qPCR array (CBAID0085RE; Qiagen, Germantown, MD, USA). The Bacterial vaginosis microbial DNA qPCR array is a PCR amplification-based method designed to target and identify bacterial 16S rRNA gene and fungal rRNA gene sequences. The conventional assay can identify 42 bacteria as well as a protozoon and fungi (from the Aspergillus and *Candida* genera). We customized our array to contain assays for 42 microbes of interest (Table 6; 37 bacteria, 1 protozoon, and 4 fungi), which were selected based on observations from our previous 16S rRNA gene amplicon surveys (7, 31) and a *priori* knowledge of their relationships with BV. While our customized assay could also identify fungal species, we did not examine any of them since we used a DNA extraction method that was not appropriate for their detection. The only STIs examined were C. trachomatis, N. gonorrhoeae, and T. vaginalis, whereas emerging sexually transmitted pathogens (pathobionts) consisted of M. genitalium, *M. hominis*, *U. parvum*, and *U. urealyticum*.

**TABLE 6:** Assay table of the customized bacterial vaginosis microbial qPCR array^*a*^

Assay no.	Gene symbol	Description	Detected in our previous 16S rRNA gene amplicon surveys (7, 31)
1	Aerococcus christensenii	Bacterium	Possible
2	Atopobium vaginae	Bacterium	Yes
3	Bacteroides fragilis	Bacterium	Possible
4	Bacteroides ureolyticus	Bacterium	Possible
5	Candida albicans	Fungus	Not applicable
6	Candida glabrata	Fungus	Not applicable
7	Candida krusei	Fungus	Not applicable
8	Candida parapsilosis	Fungus	Not applicable
9	Chlamydia trachomatis	Bacterium	Yes
10	Clostridium sordellii	Bacterium	Possible
11	Corynebacterium aurimucosum	Bacterium	Possible
12	Dialister pneumosintes	Bacterium	Possible
13	Finegoldia magna	Bacterium	Possible
14	Fusobacterium nucleatum	Bacterium	Possible
15	Fusobacterium periodonticum	Bacterium	Possible
16	Gardnerella vaginalis	Bacterium	Yes
17	Haemophilus influenzae	Bacterium	Yes
18	Lactobacillus crispatus	Bacterium	Yes
19	Lactobacillus gasseri	Bacterium	Yes
20	Lactobacillus iners	Bacterium	Yes
21	Lactobacillus jensenii	Bacterium	Yes
22	Lactobacillus vaginalis	Bacterium	Possible
23	*Leptotrichia amnionii*	Bacterium	Possible
24	Mobiluncus curtisii	Bacterium	Possible
25	Mobiluncus mulieris	Bacterium	Possible
26	Mycoplasma genitalium	Bacterium	Possible
27	Mycoplasma hominis	Bacterium	Possible
28	Neisseria gonorrhoeae	Bacterium	Possible
29	Parvimonas micra	Bacterium	Possible
30	Prevotella bivia	Bacterium	Possible
31	Prevotella disiens	Bacterium	Possible
32	Prevotella intermedia	Bacterium	Yes
33	Prevotella melaninogenica	Bacterium	Yes
34	Prevotella nigrescens	Bacterium	Yes
35	Shuttleworthia satelles	Bacterium	Possible
36	Sneathia sanguinegens	Bacterium	Yes
37	Streptococcus agalactiae	Bacterium	Yes
38	Streptococcus intermedius (1338)/Streptococcus constellatus	Bacterium	Possible
39	Streptococcus mitis	Bacterium	Possible
40	Trichomonas vaginalis	Protozoon	No
41	Ureaplasma parvum	Bacterium	Possible
42	Ureaplasma urealyticum	Bacterium	Possible
43	Human GAPDH genomic DNA	Control	Not applicable
44	Human HBB1 genomic DNA	Control	Not applicable
45	Pan Aspergillus/*Candida*	Control	Not applicable
46	Pan bacterium 1	Control	Not applicable
47	Pan bacterium 3	Control	Not applicable
48	PPC	Control	Not applicable

a 1338, taxon identifier; GAPDH, glyceraldehyde 3-phosphate dehydrogenase; HBB1, hemoglobin subunit beta-1; PPC, positive PCR control.

The status “possible” (in the last column of Table 6) means that based on our previous 16S rRNA gene amplicon surveys (7, 31), we cannot conclusively report that the microbe in question (in the column labeled “Gene Symbol”) was detected—since we did not achieve species-level classification. The deepest taxonomic level achieved for these microbes (with “possible” “status”) was genus. Thus, for such microbes, their specific identities are unknown. The status “not applicable” indicates that the feature in question was not assessed in our previous studies.

Each sample, including a no-template control (NTC) and an in-house mock community, was run alongside the following controls: pan bacterium 1, pan bacterium 3, Hs/Mm.GAPDH, and positive PCR control (PPC). The host GAPDH control detects the presence of human genomic DNA. The inclusion of “pan bacteria” served as a control to determine the presence of bacteria in a sample. The PPC was used to test for the presence of inhibitors in the sample and/or the efficiency of the PCR. The NTC was utilized to monitor any potential nucleic acid contamination (in the reagents or acquired during the experimental procedures) and primer-dimer formation that could yield false-positive results. The mock community was used to validate our assay. This mock community was DNA from a cervical sample whose expected taxonomic profile was previously estimated using 16S rRNA gene amplicon sequencing (31). In the aforenamed 16S rRNA metagenomic sequencing, all samples were run concurrently with the Human Microbiome Project (HMP) mock communities: HM-782D (even concentration) and HM-783D (staggered concentration) (BEI Resources, Manassas, VA, USA). Thus, we had a clue to the taxonomic landscape of the all the samples.

Real-time PCR was performed using a 100-ng genomic DNA. This was added to ready-to-use 2× master mix ROX (Qiagen, Germantown, MD, USA) and microbial DNA-free water. A total of 10 μL of the reaction mixture was aliquoted into each well of a plate containing predispensed primers and hydrolysis probes. Thermal cycling was performed on a QuantStudio 12K Flex real-time PCR detection system (Thermo Fisher, Singapore) as follows: an initial PCR activation step at 95°C for 10 min, followed by 40 cycles of 15 s at 95°C for denaturation and 2 min at 60°C for annealing and extension. The raw data were first transformed using universal custom PCR array patch 141512, a data analysis patch. The cycle threshold (*C_T_*) values were analyzed to identify the presence or absence of microbes using the custom PCR array template Excel file v2.0 available through Qiagen’s GeneGlobe Data Analysis Center (https://geneglobe.qiagen.com/za/analyze). A *C_T_* value of <34 and ≥34 was regarded as positive and negative, respectively, for the microbe in consideration. This was based on the *C_T_* value (40) for the NTC (which was run in every plate) and the lower *C_T_* value (6) set for a positive call. Our final analyses included only samples that were positive for any of the microbes and had all the controls passed.

### Data analysis: statistical associations and hierarchical clustering.

Statistical analyses were done using Prism v6.01 (GraphPad Software, Inc., San Diego, CA, USA). Participants’ categorical variables were summarized as percentages and frequencies, while continuous variables were expressed as medians with interquartile ranges (IQRs) at the 25th and 75th percentiles. Comparison of the variables between women aged 30 to 39 years and 40 to 49 years as well as those over 50 years old were computed using chi-square/Fisher’s exact tests, with statistical significance at a two-tailed *P* value of <0.05. The chi-square test was applied only if the expected frequencies in a 2 × 2 contingency table were ≥5 or if at least 80% of the cells in a 2 × 3 contingency table had an expected frequency of ≥5 and no cell had an expected frequency <1; otherwise, Fisher’s exact test was applied. Chi-square/Fisher’s exact tests were also used to compute the association of cumulative prevalence of STIs and pathobionts with HIV status and demographic and sexual behavior. Along with this, we tested the association of pathobionts and *Lactobacillus* spp. with HIV as well as the association between *Lactobacillus* spp. and the age group of women. A two-tailed *P* value of <0.05 was considered statistically significant. Odds ratios (ORs) with corresponding 95% confidence intervals (CIs) were used to estimate the magnitude of associations.

To identify whether the women could be grouped according to the binary data (presence and absence of cervical microbe), we performed an unsupervised hierarchical clustering. Pairwise scores between samples were computed based on the Jaccard dissimilarity index using the vegan R package v2.5 (62). Clustering was based on the average neighbor linkage method. The gap statistic method (63) was used to estimate the optimal number of clusters, with 10 as the k.max (the maximum number of clusters to consider) and 500 as the bootstrapping value. The largest gap statistic is usually considered the optimal number of clusters.

### Ethics approval.

The study was approved by the Human Research Ethics Committee (HREC) of the University of Cape Town, South Africa (HREC reference 615/2017). The study was described to all the eligible participants, and written informed consent was obtained.

### Data availability.

Data are available upon reasonable request. The deidentified data are owned by the partner institutions. Requests for data utilization should be sent to the corresponding author.
